# TelereHUB-CHILD: An online integrated knowledge translation tool to optimize telerehabilitation evidence-based practices for children with disabilities and their families

**DOI:** 10.3389/fresc.2023.1139432

**Published:** 2023-03-27

**Authors:** Tatiana Ogourtsova

**Affiliations:** ^1^ Department of Medicine and Health Sciences, School of Physical and Occupational Therapy, McGill University, Montreal, QC, Canada; ^2^ The RESI-ALLIANT KID Laboratory, Research Center of the Jewish Rehabilitation Hospital, Integrated Health and Social Services Centers of Laval, Site of Centre for Interdisciplinary Research in Rehabilitation of Greater Montreal, Laval, QC, Canada

**Keywords:** telerehabilitation, pediatrics, developmental disabilities, integrated knowledge translation, evidence based practice, patient oriented research

## Abstract

**Background:**

Pediatric telerehabilitation has been quickly adopted by clinicians during the pandemic. This precipitated shift in the model of healthcare delivery is significant and compounded by clinicians' training and knowledge needs related to evidence-based practices. This instigated a knowledge translation initiative TelereHUB-CHILD—an online platform designed for clinicians, patients, and families. The aim of this brief report is to describe its development, including the roles of key stakeholders in these processes.

**Methods:**

Following a systematic review on telerehabilitation, a series of co-creation activities with clinical (*n* = 24 rehabilitation professionals) and parent-partners (*n* = 4 parents of children with disabilities) were undertaken. Clinical partners were engaged in five web-activities. These were designed to gather their feedback regarding training and knowledge needs, present preliminary findings of the systematic review and explore their perceived importance and usefulness with respect to different sections of TelereHUB-CHILD, including *Tele-treatments*, *Tele-Assessments*, and *Resources*. Parent-partners were engaged asynchronously to provide feedback on the content and presentation of the *Patient/Family Information* section.

**Results:**

Clinical partners reported moderate-high usefulness and importance with each section of the tool and the presented features. As per partners' feedback, the *Tele-treatments* section provides standardized summaries outlining the effectiveness of the tele-treatment approach and the level of the evidence for each outcome of interest, according to the different diagnosis groups and professional discipline. For patients/family, common questions and answers can be explored in three user-friendly formats, including printable learning briefs, onsite accordions, and animation videos. The *Tele-assessments* section outlines existing measures by professional discipline. *Resources* offer preparatory forms for families and clinicians, questionnaires, and other learning material.

**Conclusion:**

TelereHUB-CHILD was co-developed with key stakeholders. It can guide telerehabilitation evidence-based practices, empower patients and families, and pinpoint research and practice gaps.

## 1. Introduction

There are 240 million children around the world living with a disability (1). Developmental disabilities such as cerebral palsy and autism spectrum disorder are chronic conditions that are associated with substantial individual and societal implications (2). Children with developmental disabilities face significant life-long challenges affecting their daily performance, participation, and quality of life (3, 4). These children and their caregivers are in high need of comprehensive rehabilitation services in order to maximize their functional potential, prevent deterioration and crises, and optimize their engagement in various life aspects (e.g., school, community, leisure). Unfortunately, evidence shows that the traditional service delivery models for this population are not standardized, nor are they nimble enough to address the complex and lasting effects of developmental disabilities (5–10).

With the COVID-19 pandemic, the need for services for this population is predicted to further rise (11, 12). In these unprecedented times, the field of telerehabilitation (Telerehab) has opened a promising window of opportunity that can improve accessibility and equity to services, promote family-centeredness, engagement in therapy, and health outcomes (13, 14). In the context of national and international research priority setting taskforces, academics, researchers, practicing clinicians, and patients have conveyed that telehealth is one of the leading avenues and top priorities in pediatric research (15, 16). Moreover, while the pandemic has demonstrated that Telerehab is a possible modality, appropriate targets for this approach must be clarified and clinician guidance on implementation are still highly needed (13, 14). This is a priority as evidence-based practice in pediatric rehabilitation is associated with better health outcomes and cost-effectiveness (17). Therefore, initiatives to develop evidence-based resources are necessary. This instigated an integrated knowledge translation project entitled TelereHUB-CHILD (TELEREhabilitation HUB for CHILDren with disabilities). TelereHUB-CHILD is a comprehensive, user-friendly, online knowledge translation platform designed with and for pediatric rehabilitation specialists, patients and families. The aim of this brief report is to describe its development, including outlining the roles of key stakeholders in this process.

## 2. Methods

### 2.1. Theoretical approach

**Figure 1:**
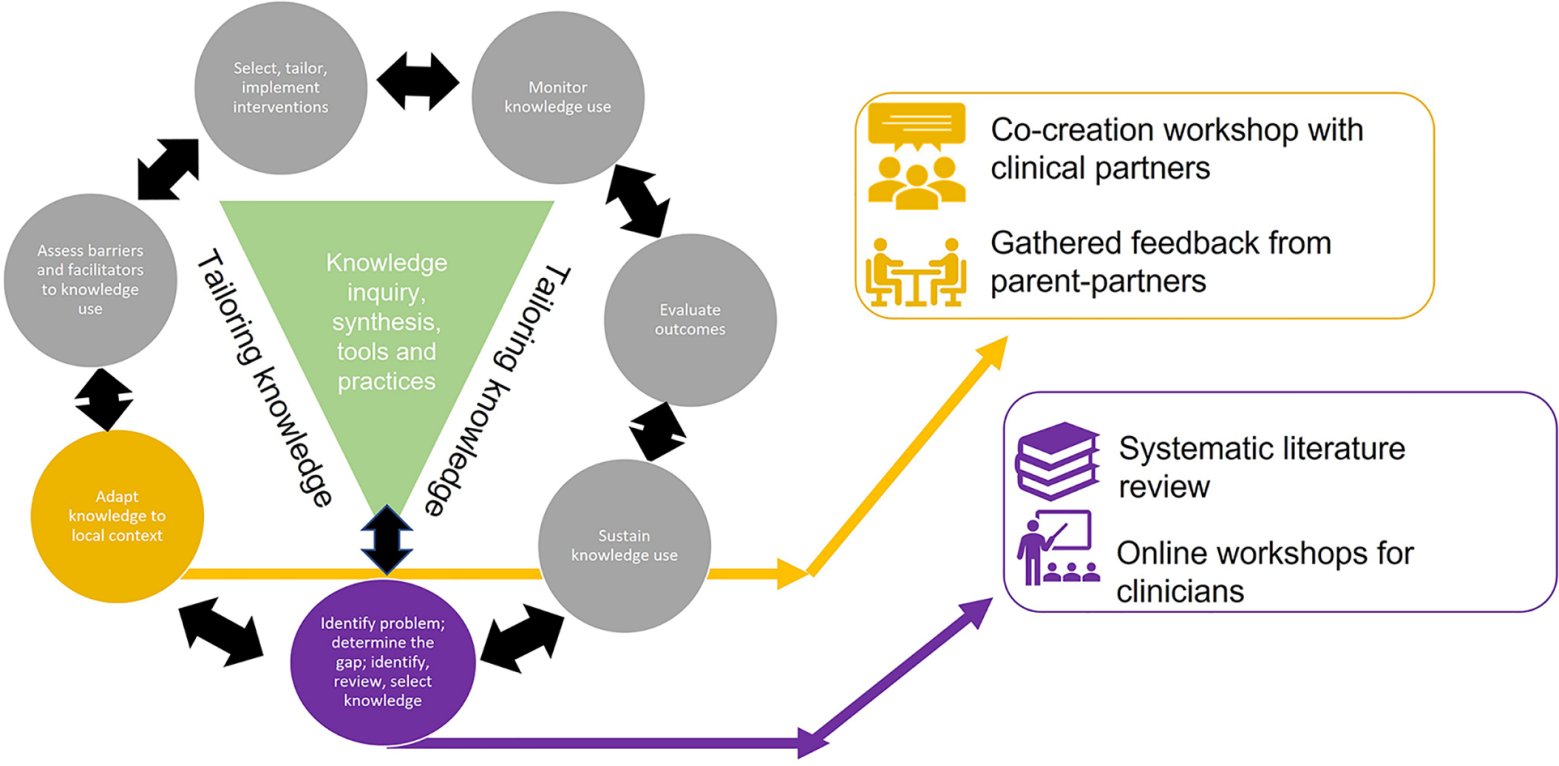
Knowledge to Action framework in the design of TelereHUB-CHILD.

The development of TelereHUB-CHILD is grounded in an integrated knowledge translation approach (iKT) (18), a robust method of co-production and KT where knowledge users are equal partners in the research process. Integrated KT is utilized to pose research questions that matter to knowledge users and generate results which are more meaningful to them, and therefore, more likely to be useful (19, 20). Furthermore, a robust process was applied to support the initiative, the Knowledge to Action (KTA) framework (21). The KTA framework provides a structured process for moving through the KT process. It includes a seven-step action cycle that guides the translation of new knowledge into practice: (1) Create knowledge; (2) Adapt to local context; (3) Assess barriers/facilitators to knowledge use; (4) Select, tailor, implement interventions; (5) Monitor knowledge use; (6) Evaluate outcomes; and (7) Sustain knowledge use (21). *Step 1—Create knowledge* and *Step 2—Adapt to local context* were applied in the development of TelereHUB-CHILD (Figure 1).

### 2.2. Study design

In *Step 1—Create Knowledge*, a systematic review on existing tele-interventions and assessments for children/youth with developmental disabilities and their families was conducted (13). In *Step 2—Adapt to local context*, a cross-sectional snapshot approach in a context of a co-creation webinar on Telerehab and collaborative development activities with parent-partners using strategies for patient-oriented research were used.

### 2.3. Partnership groups

Partnerships were established with two main stakeholder groups, including pediatric rehabilitation professionals and parents of children with disabilities. To target the rehabilitation professionals, clinical managers and clinical research coordinators from three urban pediatric rehabilitation settings providing services to children with developmental disabilities and their families (Montreal and Laval areas, Quebec, Canada) were engaged to promote and facilitate the logistics of KT activities (described in detail below). Moreover, a parent-advisory committee was formed to include parents (*n* = 4, 3 mothers, 1 father) of children (age range 18 months to 21 years old) with various developmental disabilities (e.g., autism spectrum disorder, cerebral palsy, epilepsy). Members of this advisory committee were recruited by the author's (TO) research laboratory using a snowball and word-of-mouth recruitment strategies. Parent-partners are regularly engaged in patient-oriented research activities of the laboratory since March 2021.

### 2.4. Procedures, measurement, and analysis

In *Step 1—Create Knowledge*, procedures related to the PROSPEPO-registered systematic review on pediatric Telerehab (#CRD42020204799) are described in full detail elsewhere (13). The findings of the systematic review were considered for translation into the KT tool. Namely, the review outlined evidence on tele-treatments for six distinct diagnosis groups (e.g., children with autism spectrum disorder, cerebral palsy) and for multiple (*n* > 200) child- and parent-related health outcomes. The review comprehensively reported the effectiveness of each tele-approach, the comparison intervention, and the level of the evidence. Moreover, information on the health providers' discipline (e.g., a tele-treatment provided by occupational therapists) was extracted and allowed to build another classification layer into the KT tool. In addition, the review identified several tele-assessments, along with information on its purposes and administration specifics (e.g., professional involved, equipment used, training required). Overall, these findings served to design a first draft for the KT tool prototype, including major sections (i.e., tele-treatments; tele-assessments), subsections (by diagnosis groups, by professional discipline), and other potentially useful attributes (e.g., effectiveness of the approach, levels of evidence for each outcome, comparison intervention).

Furthermore, TelereHUB-CHILD's prototype was inspired from an existing and well-established online KT tool, StrokEngine (www.strokengine.ca). StrokEngine provides evidence-based information for clinicians and patients/families on stroke rehabilitation strategies and has been shown to be highly usable and navigable among rehabilitation professionals (22). Moreover, laypersons reported being overall satisfied with the patient/family modules of StrokeEngine and reported that the platform is easy to use and is valuable in content (23). The author (TO) has developed over fifteen (*n* = 15) StrokEngine learning modules [e.g., Aerobic exercise (24)], including sections for clinicians and for patients/families. This experience has been applied by the author in designing the KT modules skeletons for TelereHUB-CHILD.

In *Step 2—Adapt to local context*, partnerships with pediatric rehabilitation professionals were established and fostered through the luncheon series entitled *Telerehabilitation Luncheon Seminars—as a way to exchange & co-develop roadmaps towards effective use of telerehabilitation*. These online luncheon series were delivered in three parts between Dec 2020–2021: PART 1- Getting to know each other & share experiences (*n* = 1 workshop); PART 2—On the way towards effective use of telerehabilitation (*n* = 3 workshops), and PART 3—Co-creation of knowledge translation tools (*n* = 1 final workshop).

• In PART 1, the plan for the roadmap was introduced to attendees (*n* = 51 clinicians, *n* = 17 occupational therapists, *n* = 11 physical therapists, *n* = 9 speech language pathologists, *n* = 7 social workers, *n* = 3 vision specialists, *n* = 2 special educators, *n* = 1 psychoeducator, *n* = 1 clinical coordinator). It consisted of presenting the research team, outlining the timeline, activities, and foreseeable outputs. Moreover, using the breakout rooms, clinicians were given the opportunity to informally exchange about their experiences with Telerehab, what hinders (i.e., existing barriers), and what enables (i.e., existing facilitators) their Telerehab practices. The major points raised in these exchanges were discussed in the main meeting once participants reconvened.
• In PART 2, two (*n* = 2/3) workshops were presented online to pediatric clinicians (*n* = 40, *n* = 34 respectively). These workshops aimed to outline the preliminary findings of the systematic review on tele-treatments. One workshop (*n* = 1/3) was pre-recorded and focused on tele-assessments that were identified through the systematic review. A link to the recording was sent to the clinical coordinators at the participating sites, along with discipline specific information briefs in a PDF format (e.g., tele-assessments in physical therapy; tele-assessments in speech language pathology).
• In PART 3, clinicians were invited to engage in the last workshop: a knowledge exchange and collaborative building activity. In this workshop, the prototype for the TelereHUB-CHILD was presented. It consisted of the following sections: (1) *Tele-treatments: Clinician Information*, *Patient/family Information*; (2) *Tele-assessments*; (3) *Resources*. Using the polling feature in Zoom, clinical partners (*n* = 24; *n* = 11 speech language pathologists; *n* = 10 occupational therapists; *n* = 2 special educators; and *n* = 1 physical therapist) were asked to rate the different features of each section for their importance and/or usefulness (described in detail below). A four-point Likert scale ranging from *Not useful / important at all* to *Very useful/important* was employed.

Parent-partners were engaged separately from clinical partners. A prototype of a *Patient/family Information* section was designed for one of the six diagnosis groups (autism spectrum disorder) following a question-and-answer format, previously shown to be useful, usable and understandable by laypersons in the context of a KT tool in rehabilitation (23). This draft was sent to the parent-partners and their feedback was requested on the completeness, appropriateness, and understandability of the content. Parent-partners were offered the option to provide comments/made edits directly on the created prototype using the Track Changes option in Microsoft Word and/or provide written feedback via email. A period of two weeks was allotted to complete this activity.

#### 2.4.1. *Tele-Treatments* section of TelereHUB-CHILD

The elements to be rated in the section on *Tele-Treatments: Clinician Information* included:

• All content subdivision by diagnosis groups (e.g., autism spectrum disorder vs. cerebral palsy)
• All content subdivision by clinical discipline (e.g., occupational therapy vs. psychology)
• Content and presentation of the introduction/overview statement:
  ○ Outline of the included studies (e.g., randomized clinical trial of high quality, quasi-experimental study design)
  ○ Outline of intervention target (e.g., parent vs. child-targeted) and focus (e.g., to improve motor abilities vs. behavior)
  ○ Outline of the used platform (e.g., Zoom vs. Skype vs. phone/chat)
  ○ Outline of clinician involvement (e.g., active in all session vs. monitoring intermittently)
  ○ Outline of effectiveness (e.g., more vs. as effective).
• Content and presentation of Telerehab effectiveness section:
  ○ View studied outcomes per professional discipline
  ○ View all studied outcomes
  ○ View the effectiveness for each studied outcome (e.g., more vs. as effective)
  ○ View the comparison intervention (e.g., usual care)
  ○ View the level of evidence (e.g., Level 1b—Moderate)
  ○ Ability to expand on each outcome to into a more detailed evidence-based standardized summary; and the content of the evidence-based standardized summary.

#### 2.4.2. *Patient/family information* section of TelereHUB-CHILD

The design and content features to be rated in the *Patient/family Information* section included:

• Content subdivision by diagnosis group
• Question and answer format
• Co-development process with parent-partners.

#### 2.4.3 *Tele-Assessments* section of TelereHUB-CHILD

The features to be rated in the *Tele-Assessments* section were comprised of:

• Content subdivision by clinical discipline
• Information on:
  ○ Assessment's purpose (e.g., to evaluate motor functions vs. visual perception)
  ○ Eligible population (e.g., employed in children with cerebral palsy)
  ○ Equipment and platform used
  ○ Face-to-face comparison
  ○ Findings of the study (e.g., agreement results between tele-method and face-to-face assessment method, psychometric properties)
  ○ Ability to see measure or a link to measure provider
  ○ Training requirements for the healthcare professional.

#### 2.4.4. *Resources* section of TelereHUB-CHILD

The features to be rated in *Resources* section were:

• Discipline specific forms to prepare patients and families for Telerehab visits
• Resources to evaluate client and/or clinical settings' potential for Telerehab
• Information on confidentiality, ethics, data storage and charting
• Integrated surveys aiming to further improve Telerehab practices
• Discussion board for clinicians and patients/families to share experiences
• Training modules in form of short videos
• Training modules in form of case-based learning and quizzes to assess knowledge gained.

### 2.5. Results

**Figure 2:**
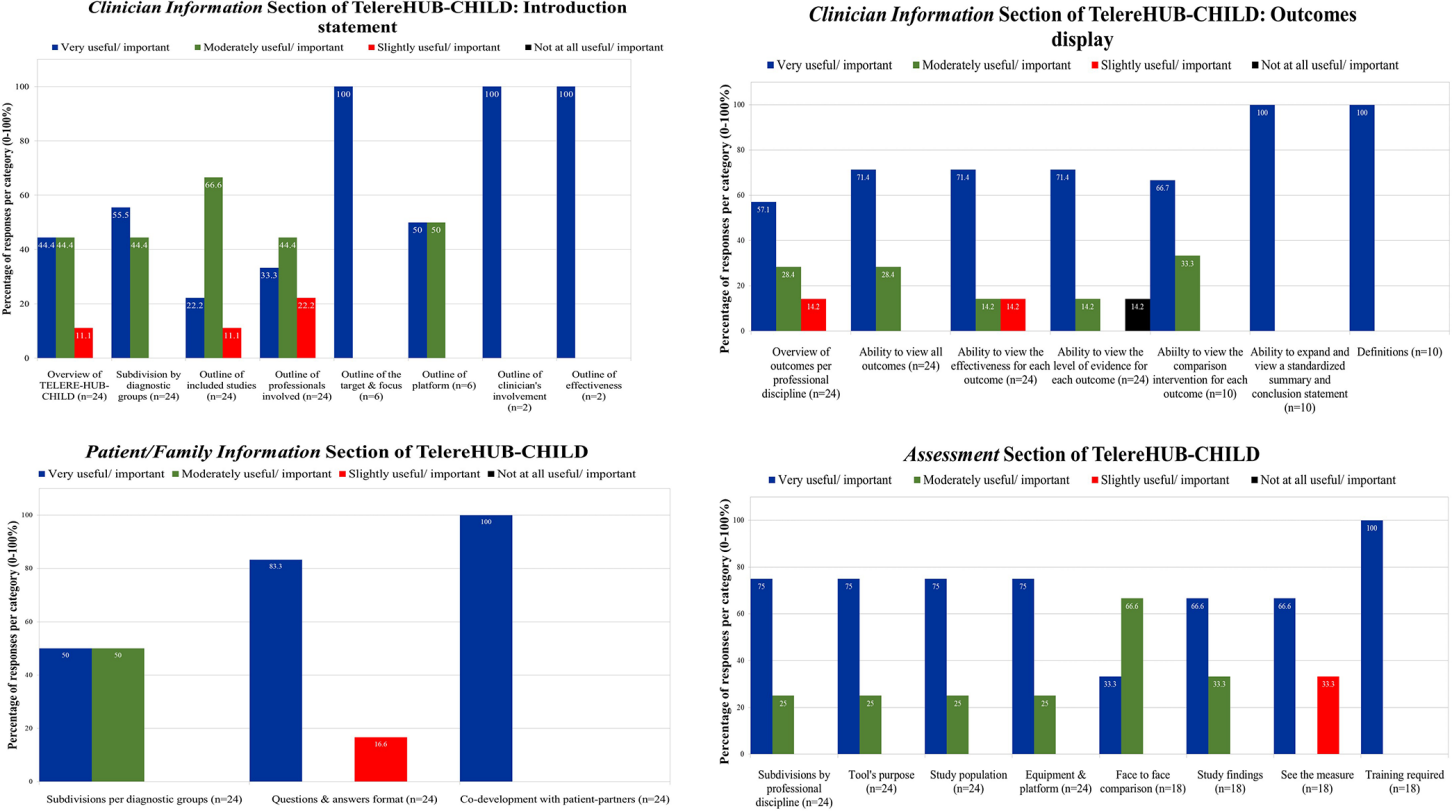
Importance and usefulness ratings of clinical partners on the Tele-Treatment and Tele-Assessment sections of TelereHUB-CHILD.

**Figure 3:**
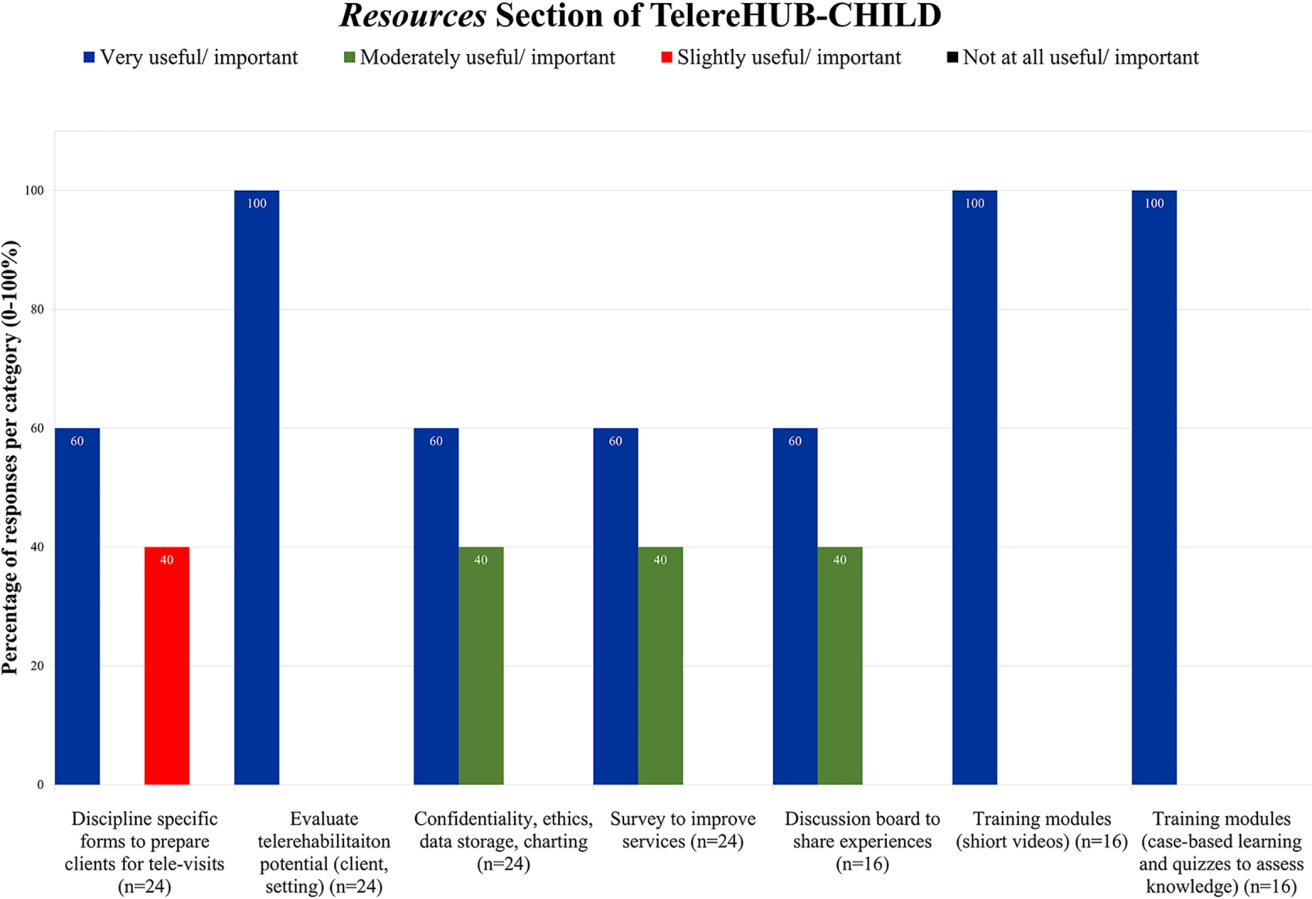
Importance and usefulness ratings of clinical partners on the Resources section of TelereHUB-CHILD.

The responses of clinical partners are outlined in Figures 2, 3.

#### 2.5.1. *Tele-Treatments* section of TelereHUB-CHILD

Overall, all (100%) clinical partners reported that subdividing the content by diagnosis groups (e.g., autism spectrum disorder vs. cerebral palsy) was moderately to very useful/important. Many (79.5%) indicated that the subdivision by professional discipline (e.g., physiotherapy vs. speech language pathology) is moderately to very useful/important. For the introduction statement (Figure 2), clinicians reported moderate to high usefulness of providing the overview of TelereHUB-CHILD (88.8%), the outline of included study designs (88.8%), professionals involved (77.7%), and platforms used (100%). Moreover, all (100%) agreed that outlining the target and focus of the tele-intervention, the clinician's involvement, and the effectiveness of the approach is very useful and important.

As per these results, the *Tele-Treatments* section was set to display the content by diagnosis group and professional discipline. Moreover, the introductory statement was set to include all the aforementioned features. TelereHUB-CHILD contains six (*n* = 6) distinct *Tele-Treatments* modules as per the following diagnosis groups: (1) autism spectrum disorder; (2) attention deficit and hyperactivity disorder; (3) cerebral palsy; (4) intellectual, speech and learning disabilities; (5) mixed diagnoses (i.e., studies including children and/or youth with various conditions); and (6) traumatic brain injury. Within each module, there are 6, 4, 5, 3, 1, and 4 submodules (for clinicians) respectively that are displayed by professional discipline.

For the display of the outcomes (Figure 2), 75.5%–100% of clinical partners conveyed moderate to high importance and usefulness in viewing outcomes per professional discipline and the ability to view all outcomes studied at once. Similarly, 85.8%–100% of them reported moderate to high importance to view the effectiveness for each outcome, the level of the evidence, the ability to expand on each outcome in order to have access to a more detailed standardized evidence-based summary and having a section on definitions/terms that are commonly employed throughout the tool (e.g., level of evidence).

As per this feedback, the display of the outcomes was set to appear as an accordion, where the user could view all outcomes at once for a particular submodule (e.g., occupational therapy, autism spectrum disorder, Supplementary Figure S1). Within that accordion, the following information was included for each outcome: level of effectiveness, comparison intervention, and level of evidence. The tool was further designed for the user to be able to easily expand on the outcome of interest to find a standardized evidence-based summary which provides more details about the study(ies) and the results (Supplementary Figure S2). Moreover, users have access to a printable PDF results tables for each submodule that provide more descriptors about each tele-intervention in terms of the studied sample, platform/equipment used, frequency and duration, target and focus, as well as the results. These results tables were designed for each diagnosis group and per professional discipline. Overall, across all modules, standardized evidence-based summaries are outlined for two-hundred and three (*n* = 203) outcomes, ranging from sixty-three (*n* = 63, autism spectrum disorder module) to eleven (*n* = 11, mixed module) summaries.

#### 2.5.2. *Patient/family information* section of TelereHUB-CHILD

For this section, 83.3%–100% of the group indicated moderate to high importance and usefulness to subdivide the content per diagnosis group, to present the content in a questions and answer format and to co-develop this section in collaboration with parent-partners (Figure 2). As such, six *Patient/family Information* modules were designed (one for each diagnosis group), following a question/answer format with twelve (*n* = 12) questions and answers per module. Examples of questions are: “*Is this appropriate for me and my child?*” and “*How many treatments are necessary to make progress?*”.

Following the review and feedback received from parent-partners, in addition to the information being displayed in reading format, a printable PDF and a short Animaker® video were also created for each module to accommodate different learning needs. In the video, a parent of a child is asking questions about Telerehab to a healthcare professional who is providing the answers with supporting visual material. Moreover, parent-partners have provided feedback for the question *“How many treatments are necessary to make progress?”.* Specifically, it was suggested to incorporate caregivers’ perspective and a collaborative meaning into the answer as follows: *“In collaboration with your rehabilitation professional, you can determine the duration and frequency of tele-treatments that are most optimal and suitable for you and your child.”.* In addition, parent-partners suggested adding examples to health-related outcomes to facilitate understand [e.g., ability to process sensory information (e.g., sounds, movement, textile)].

#### 2.5.3. *Tele-Assessments* section of TelereHUB-CHILD

For this section, 100% of clinicians indicated moderate to high importance and usefulness for all features except “See the measure”, where 66.6% of participants indicated a moderate importance (Figure 2). In result, six (*n* = 6) *Tele-Assessments* modules were designed per professional discipline, including: (1) Audiology; (2) Multidisciplinary team; (3) Neuropsychology/psychology; (4) Physiotherapy; (5) Speech language pathology; and (6) Vision. Within each *Tele-Assessment* module, submodules (*n* = 17 in total, range: 1–6) are listed to display the different outcomes that were assessed (e.g., signs and symptoms of autism spectrum disorder in Multidisciplinary team, visual acuity in Vision). As in the section on *Tele-Treatments* outcomes and *Family/patient Information*, an accordion was designed to display the different features of the assessment that could be clicked on and explored further (e.g., population, face-to-face comparison, Supplementary Figure S3).

#### 2.5.4. *Resources* section of TelereHUB-CHILD

For this section, 60%–100% of the partners indicated high importance in having discipline specific forms to prepare patients and families for visits, having access to resources evaluating Telerehab potential and training modules (in form of videos and case-based learning). Moderate (in 40% of respondents) to high (60% of respondents) usefulness was reported for resources about confidentiality, ethics, data storage, charting; surveys to improve services; and discussion board where experiences could be shared (Figure 3).

In view of these findings and additional feedback from parent-partners, the *Resources* section was designed to have two modules: one for patients/family (https://telerehubchild.com/resources-for-patients-and-families/) and one for clinicians (https://telerehubchild.com/resources-for-clinicians/). The patient/family module contains supporting material on how to get ready for Telerehab, including advice for different situations regarding wi-fi and device access, items and space/environment needed, and tips and tricks for comfort and privacy (e.g., for teens). Downloadable and fillable PDFs are available for patients and family to further prepare for a Telerehab visit (https://telerehubchild.com/wp-content/uploads/2023/02/PIF_Feb19_1-1.pdf), and checklists to ensure readiness depending on wi-fi access at home (https://telerehubchild.com/wp-content/uploads/2023/02/WAH_EN_Feb19_5-5.pdf) or remotely (https://telerehubchild.com/wp-content/uploads/2023/02/RAW_EN_Feb19_7-7-1.pdf).

For clinicians, the *Resources* section includes information on how to access the settings' and patient's Telerehab readiness, a training video on levels of evidence, and a fillable PDF form to prepare patients and families for a Telerehab visit (https://telerehubchild.com/wp-content/uploads/2023/02/CF_EN_Feb24-1.pdf). This form is discipline specific, where a clinician can indicate the purpose (i.e., assessment, treatment), target (e.g., parent vs. child), and focus of the visit (e.g., to work on child's speech and language abilities and parental self-efficacy); and items required.

## 3. Conclusion

The aim of this brief report was to describe the co-creation process of an iKT tool for pediatric telerehabilitation, TelereHUB-CHILD, and to outline the roles of the key stakeholders in this initiative. In a series of collaborative activities with key stakeholders and end-users, the content of TelereHUB-CHILD and its display were adjusted to reflect partners' feedback. Overall, most prototype's features were moderately to highly rated for importance and usefulness by clinical-partners and were therefore included in the product to reflect these ratings. The materials reviewed by parent-partners for the *Patient/family Information* and *Resources* sections were adjusted as per their feedback and additional modalities of the material were created to suit a set of diverse learning needs (e.g., visual vs. auditory learning, on- vs. offline learning).

TelereHUB-CHILD directly addresses the reported need for training in telerehabilitation evidence-based approaches previously reported by pediatric health providers (14). The created online platform has multiple benefits. First, a pediatric rehabilitation clinician can find vetted and evidence-based information on what works, for whom, and how. In addition, the levels of evidence for the obtained results can facilitate their decision making with regards to the clinical implementation of a strategy; therefore, it could serve to optimize and enhance the use of evidence-based rehabilitation. For a caregiver of a child with disability or youth with disability, this online platform could empower them with knowledge and the needed resources; thus, potentially fortifying health-related outcomes and therapy engagement for themselves and/or their child. For academics and researchers, this tool could be used to pinpoint missing areas and gaps in research and practice that necessitate further and/or more high-quality work.

Online KT tools in the field of childhood disability have been launched previously. For instance, the Peer Support Best Practice Toolkit is an evidence-informed resource synthesizing best practices in peer support for program providers working with families of children with disabilities and complex medical needs (25). Its early proximal indicators such as web traffic are shown to be promising (26). The Childhood Disability LINK is another example of an online knowledge mobilization initiative in the field of childhood disability, with a focus on different therapies, new knowledge, leisure, family support, and policy (27). Recently, Childhood Disability LINK launched EDIT-CP (Early Detection and Intervention Toolkit for Cerebral Palsy (https://www.childhooddisability.ca/edit-cp-toolkit/)) (28). This online iKT toolkit aims to promote and enhance early detection and intervention in children with cerebral palsy. The early intervention section of EDIT-CP follows a similar format to the TelereHUB-CHILD in terms of the levels of evidence being available for studies outcomes and specific modules for patients/families. The usability and the impact of EDIT-CP are yet to be determined (29). Overall, it emerges that TelereHUB-CHILD is unique in addressing an existing gap in mobilizing knowledge about pediatric Telerehab into clinicians' and families' hands.

Given that the platform was designed in tight collaboration with end-users, the way in which the content is presented is more likely to be adopted and satisfactory. For instance, in the co-creation webinar, it was made clear by clinical partners that they wish to see all the studied outcomes at once (per diagnosis group and per professional discipline) and then be able to choose the ones of interest to them so that they could expand on it to find out more. Hence, the accordion presentation of the outcomes was designed to reflect that need, where in each accordion line, a user can quickly view the outcome of interest, the effectiveness, comparison interview, and the level of evidence.

The present initiative has limitations. Namely, the co-creation process did not include youth with disabilities, which could have been highly appropriate provided that certain tele-interventions targeted adolescents. Moreover, although not all features were rated by all clinical partners present at the co-creation webinar, 70% of the features were rated by more than 50% of participants. Lastly, TelereHUB-CHILD is currently only available in English. However, work is ongoing to have it fully translated into French, as well as adapting resources for patients/family (e.g., preparation sheet) for the Indigenous community.

Future work with TelereHUB-CHILD is in the planning. To continue integrating partners' feedback, sections on training for clinicians, discussion boards, and information on confidentiality, ethics, data storage, and charting will be included in the *Resources* section. Moreover, to move further in the KTA process, I intend to assess barriers/facilitators to knowledge use (Step 3 of the KTA). In addition, the training material for clinicians is projected to be integrated into a standardized multimodal KT activity that could be delivered to different clinical settings worldwide (Step 4 and 5 of the KTA—Select, tailor, implement interventions; Monitor knowledge use). The impact of the training could then be investigated (Step 6—Evaluate outcomes) and the platform further enhanced to promote learning and usage (Step 7—Sustain knowledge use). In addition, there is intention to track more in-depth users' activity on the platform (i.e., navigation pathways, time spend in different section) and to integrate a visitor's survey. The survey will allow to determine users' characteristics (e.g., clinician vs. parent of a child with disability), their needs, and the impact of the platform (e.g., were they able to find what they are searching for). Moreover, yearly updates of TelereHUB-CHILD's content with newly emerged evidence that is rapidly growing worldwide is a priority.

In conclusion, TelereHUB-CHILD is an iKT initiative built in collaboration with key stakeholder groups and a thorough review/appraisal/evidence extraction process. TelereHUB-CHILD could be a valuable tool in empowering and guiding clinicians in their evidence-based Telerehab clinical practice, as well as patients and families in their rehabilitation journeys.

## Data Availability

The original contributions presented in the study are included in the article/**Supplementary Material**, further inquiries can be directed to the corresponding author/s.
