# Supplementary figures and images for: TelereHUB-CHILD: An online integrated knowledge translation tool to optimize telerehabilitation evidence-based practices for children with disabilities and their families
Source: Front Rehabil Sci. 2023 Mar 27;4:1139432. doi: 10.3389/fresc.2023.1139432 (PMC10083307; doi:10.3389/fresc.2023.1139432)

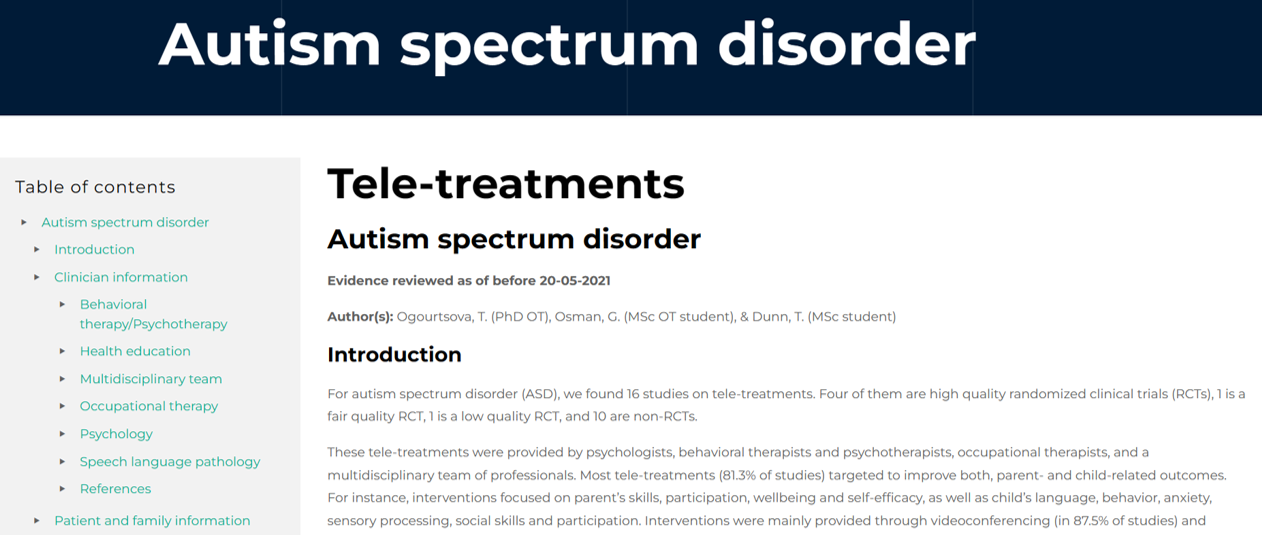

Supplement: Supplementary file 1 [file Image1.png]

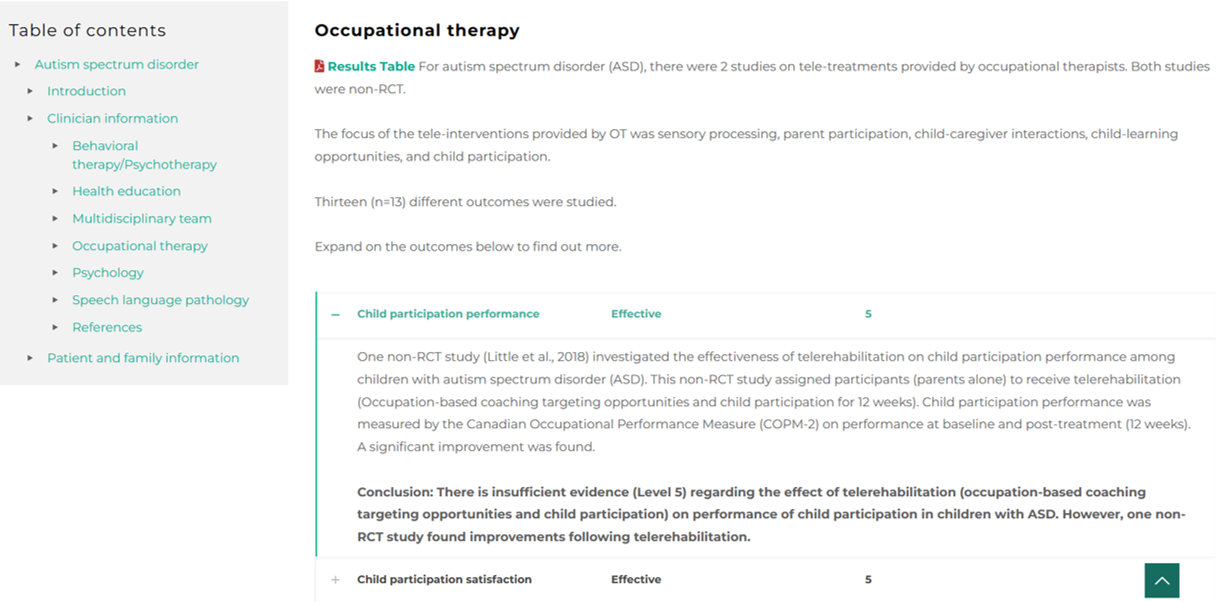

Supplement: Supplementary file 2 [file Image2.png]

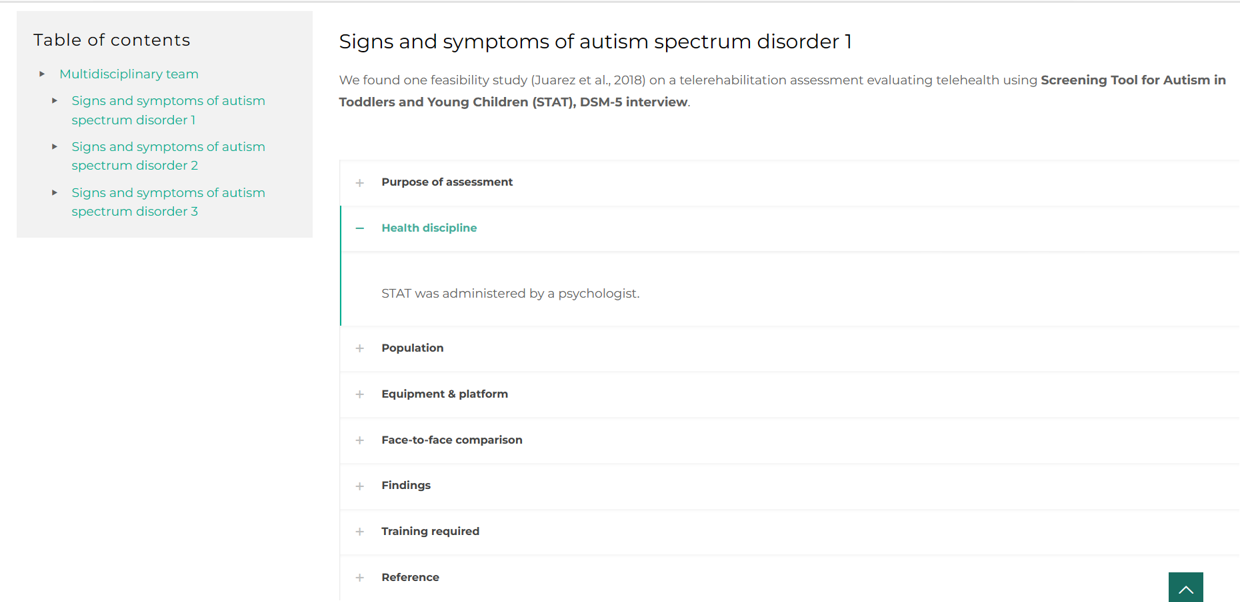

Supplement: Supplementary file 3 [file Image3.png]
